# Circulating miRNAs, isomiRs and small RNA clusters in human plasma and breast milk

**DOI:** 10.1371/journal.pone.0193527

**Published:** 2018-03-05

**Authors:** Mercedes Rubio, Mariona Bustamante, Carles Hernandez-Ferrer, Dietmar Fernandez-Orth, Lorena Pantano, Yaris Sarria, Maria Piqué-Borras, Kilian Vellve, Silvia Agramunt, Ramon Carreras, Xavier Estivill, Juan R. Gonzalez, Alfredo Mayor

**Affiliations:** 1 ISGlobal, Barcelona Ctr. Int. Health Res. (CRESIB), Hospital Clínic—Universitat de Barcelona, Barcelona, Spain; 2 ISGlobal, Centre for Research in Environmental Epidemiology (CREAL), Barcelona, Spain; 3 Genomics and Disease Group, Bioinformatics and Genomics Program, Centre for Genomic Regulation (CRG), Barcelona, Spain; 4 Universitat Pompeu Fabra (UPF), Barcelona, Spain; 5 CIBER Epidemiología y Salud Pública, Barcelona, Spain; 6 Harvard TH Chan School of Public Health, Boston, MA, United States of America; 7 Microarray Analysis Service, IMIM (Hospital del Mar Medical Research Institute), Barcelona, Spain; 8 Laboratory of Childhood Leukemia, Department of Biomedicine, University of Basel and Basel University Children's Hospital, Hebelestrasse, Basel, Switzerland; 9 Obstetrics and Gynaecology Department, Hospital del Mar, Parc de Salut Mar, Barcelona, Spain; 10 Pediatrics, Obstetrics and Gynecology and Preventive Medicine Department, Universitat Autònoma de Barcelona, Barcelona, Spain; 11 Genetics of Child and Woman's Health Group, Research Department, Sidra Medical and Research Center, Doha, Qatar; 12 Genetics Unit, Dexeus Woman's Health, Barcelona, Spain; 13 Centro de Investigação em Saúde da Manhiça (CISM), Maputo, Mozambique; University of Texas MD Anderson Cancer Center, UNITED STATES

## Abstract

Circulating small RNAs, including miRNAs but also isomiRs and other RNA species, have the potential to be used as non-invasive biomarkers for communicable and non-communicable diseases. This study aims to characterize and compare small RNA profiles in human biofluids. For this purpose, RNA was extracted from plasma and breast milk samples from 15 healthy postpartum mothers. Small RNA libraries were prepared with the NEBNext® small RNA library preparation kit and sequenced in an Illumina HiSeq2000 platform. miRNAs, isomiRs and clusters of small RNAs were annotated using seqBuster/seqCluster framework in 5 plasma and 10 milk samples that passed the initial quality control. The RNA yield was 81 ng/mL [standard deviation (SD): 41] and 3985 ng/mL (SD: 3767) for plasma and breast milk, respectively. Mean number of good quality reads was 4.04 million (M) (40.01% of the reads) in plasma and 12.5M (89.6%) in breast milk. One thousand one hundred eighty two miRNAs, 12,084 isomiRs and 1,053 small RNA clusters that included piwi-interfering RNAs (piRNAs), tRNAs, small nucleolar RNAs (snoRNA) and small nuclear RNAs (snRNAs) were detected. Samples grouped by biofluid, with 308 miRNAs, 1,790 isomiRs and 778 small RNA clusters differentially detected. In summary, plasma and milk showed a different small RNA profile. In both, miRNAs, piRNAs, tRNAs, snRNAs, and snoRNAs were identified, confirming the presence of non-miRNA species in plasma, and describing them for the first time in milk.

## Introduction

Discovery of novel biomarkers for early detection and improved prognosis in non-communicable and infectious diseases is a research priority. A good biomarker should be suitable to diagnose the disease, predict its progression or regression, or to monitor the outcome after treatment and, ideally, should be easily obtained with minimum invasion [1].

Promising classes of molecular biomarkers are small RNAs, and special attention has been given to microRNAs (miRNAs) [2]. miRNAs are short (21–23 nt), single-stranded, non-coding RNAs that regulate gene expression and play essential roles in fundamental biological processes. Recent studies have shown the involvement of miRNAs in various aspects of major chronic diseases including bronchial asthma and diabetes, as well as in regulation and induction of senescence, brain function and cancer [3–8]. Changes in miRNA expression can be triggered by environmental factors such as exposure to pesticides, heavy metals, air pollution, bisphenol A and cigarette smoking [9, 10]. The content of miRNAs is also influenced by host-pathogen interactions, as has been shown for bacteria, viruses and apicomplexan parasites [11].

miRNAs can be actively secreted to circulation packaged in microvesicles, exosomes, or associated to RNA-binding proteins, such as Ago2, but cell death or apoptosis can also release miRNAs into the circulation [9]. Their resistance to pH variations, their stability during long term storage and repeated freeze-thaw cycles [12], and the fact that they can be detected in circulation but released by distant organs constitute them as potential non-invasive biomarkers to monitor the body’s pathophysiological status. However, besides some particular cases where circulating miRNAs have been tested in clinical trials [13, 14], there is lack of reproducibility between studies, highlighting the need of a standardization of laboratory and bioinformatic methods [15].

Next generation sequencing (NGS) allows not only the quantification of known miRNAs, but also the identification and quantification of novel miRNAs, isomiRs (miRNA variants) [16], and other small RNA species that can be functionally relevant in diseases and therefore used as potential disease biomarkers [17, 18]. However, only few studies have investigated circulating non-miRNA small RNAs, focusing on plasma [19, 20], urine and saliva [21] Although there is an extensive literature on the potential effects of breast milk miRNAs on offspring development [22–24], none of these previous studies has evaluated non-miRNA small RNAs in human milk.

The vast majority of small RNA bioinformatic tools are designed for the analysis of miRNAs. They usually discard multi-mapping reads derived from non-coding RNAs with duplication events in the genome, or alternatively, they count them several times or split the reads in all the genomic positions, which bias the estimations. Recently, Pantano et al. developed SeqCluster, a method for the accurate quantification of small RNAs, based on the estimation of clusters of highly similar sequences irrespective of their genomic annotation [25, 26].

This study aims to characterize miRNAs, isomiRs and small RNA clusters in plasma and milk as well as compare their profiles. For this purpose, miRNAs, isomiRs and small RNA clusters were analysed in samples collected from 15 healthy women at 24-72h post-delivery. In order to facilitate future studies in the field, detailed information on sample collection, processing and bioinformatics analysis is given.

## Materials and methods

### Study population

Fifteen Spanish healthy volunteers were enrolled in the study. Exclusion criteria were as follows: HIV, Hepatitis B or Hepatitis C positivity; severe preeclampsia; gestational diabetes and preterm delivery (<37 weeks of gestation). Basic demographics of the women and data about gestation and delivery were collected. Peripheral blood was collected at 0–48 h post-partum for 15 participants; and breast milk was collected at 48–72 h post-partum for 10 of the participants. Participants signed an informed consent and protocols were approved by the Hospital del Mar and Hospital Clinic (Barcelona, Spain) ethical committees.

### Collection and pre-processing of samples

#### Plasma

Twelve mL of peripheral blood were collected in VACUETTE® EDTA Tubes (K3EDTA vacutainers) (Greiner Bio-One, Cat No.: 456038). EDTA blood tubes were gently mixed by inverting 10 times and stored at 4°C upright until centrifugation. Plasma separation was performed by centrifugation of blood samples in horizontal rotor for 10 minutes at 2000 *x g* at 4°C. A second centrifugation at 16,000 *x g* for 15 minutes at 4°C, was performed to eliminate any cell debris. Plasma was transferred to a new RNase-free cryotube avoiding the collection of any cell debris and stored at -80°C.

Plasma haemolysis was evaluated by spectrophotometry using a NanoDrop ND-2000 equipment (Thermo Fisher Scientific). Absorbance at 414 nm of 0.2 was set as cut-off haemolysis as described before [27].

#### Milk

Fifteen mL of breast milk were extracted using an electric pump (Medela, Cat No.:008.0176) following manufacturer’s instructions and transferred to a 100 mL sterile RNase-free recipient. Milk was kept in ice and vortexed gently before centrifugation at 2000 *x g* for 10 minutes at 4°C to remove milk fat globules, cells, and large debris. Immediately after carefully transferring, supernatant was carefully transferred into new 2 mL RNase-free tubes, a second centrifugation at 16,000 *x g* for 15 minutes was performed at 4°C to remove residual fat, cell debris and the casein fraction. Skim milk, avoiding fat or cells, was transferred into 2.5 mL cryotubes and stored at -80°C.

### RNA extraction, quantification and quality control

Small RNAs were extracted using the miRNeasy Serum/Plasma kit (Qiagen, Cat No.: 217184) with minor modifications. RNA extraction was performed from at least 3 mL of plasma and milk. One mL of biofluid was filtered per each column and washed two times with ethanol 80% before elution. Each sample was measured for RNA quantity using Fluorometric quantification (Qubit™ 2.0 Fluorometer, Thermo Fisher Scientific) with the Qubit miRNA assay (Thermo Fisher Scientific) and for integrity and quality using two different methods: spectrophotometer (NanoDrop™ 1000, Thermo Scientific) and electrophoresis and flowcytometry (Agilent RNA 6000 pico chip and Agilent 2100 Bioanalyzer, Agilent Technologies).

### Library preparation and sequencing

Libraries were prepared using the NEBNext® Small RNA Library Prep Set for Illumina® (Multiplex Compatible) (NEB, Cat No.: E7330) following manufacturer instructions and using 150ng of RNA obtained from plasma samples and 500 ng from milk. Library size selection was done using acrylamide gels. Quality and concentration of cDNA libraries was checked, and finally groups of 13–20 samples were pooled at the same concentration before sequencing in an Illumina HiSeq2000 Sequencing System (50 nt, single read).

### Bioinformatic and statistical analyses

#### Quality control

The bioinformatic pipeline consisted of several steps (S1 Fig). An initial quality control (QC) was conducted using FASTX-Toolkit and FastQ Screen. After adaptor removal, reads with the following features were removed: 1) Reads <18nt, 2) Mean PHRED scores < 30, and 3) Low complexity reads based on mean score of the read. Then, QCed reads were mapped to miRNAs, and miRNA complexity was estimated as the number of miRNA genes that are observed as a function of the number of miRNA reads.

#### Identification and quantification of small RNAs

Sequences fulfilling QC were used as the input for the seqBuster/seqCluster tool that retrieves three matrices of counts: i) miRNA, ii) isomiRs, and iii) small RNA clusters [25, 28]. In order to detect miRNAs and isomiRs, reads were mapped to hairpins released from miRBasev21 using miraligner allowing one mismatch [28]. Sequences mapping ambiguously to several positions of the genome were filtered out. Only isomiRs with 3’ or 5’ trimming or 3’ addition modifications are kept in the result files. isomiRs with one nucleotide substitution are not reported due to the difficulty of linking them unambiguously to a particular miRNA. IsomiR naming followed the miRTop project’s recommendations (http://mirtop.github.io/). IsomiR names can be merged with isomiR sequences using a GFF3 format file provided on GEO. In parallel, sequences were mapped to hs37d5 using bowtie [29], and hotspots (sets of overlapping sequences according to their position in the genome) were detected. Hotspots sharing any sequence were grouped in primary clusters. Then, a recursively heuristic algorithm based on reduction and cluster correction was applied to the ones sharing over 60% of the sequences. These were annotated to different RNA species using miRBase v20 [30], refGene (RefSeq genes), wgRna (CD and H/ACA Box snoRNAs and miRNAs from Weber and Griffiths-Jones), rmsk (Repeating elements created using RepeatMasker) and tRNA from University of California Santa Cruz (UCSC) genome browser (hg19) [31], and piR_hg19_v1.0 from the regulatoryrna database (www.regulatoryrna.org) [32]. Clusters can be considered as unique units of transcription, regardless of their annotation to one or multiple positions in the genome (irrespective of their genomic origin).

Raw FASTQ files, quality controlled unnormalized counts, metadata and a GFF3 format file with isomiR annotation can be downloaded from Gene Expression Omnibus (GEO): GSE107524.

#### Normalization and differential expression

Normalization and differential expression was performed with the R package DESeq2 v.1.10.1 (R version 3.3.2) [33]. DESeq2 performs an internal normalization where geometric mean is calculated for each gene across all samples (scaling factor method) The impact of main technical and demographic/biological variables on normalized counts was explored through Principal Component Analysis (PCA) and dendrograms implemented within the FactoMineR R package [34]. Differential expression was assessed with negative binomial generalized linear models adjusting for multiple testing with the False Discovery Rate (FDR) method [35]. All models considered biofluid as the independent variable and were adjusted for subject as a fixed effect. In addition, a sensitivity analysis considering only paired samples was performed and results did not change substantially.

Milk samples presented systematically higher sequencing depth than plasma samples. As sensitivity analyses, we repeated the miRNA differential analyses with different filtering approaches. Our main analysis used DESeq2 to filter out features having little chance of showing significant evidence as they contain outliers or low mean normalized counts. Second, to avoid comparison of miRNAs not detected due to the lower sequencing depth in plasma samples, an initial filtering of miRNAs present in <80% of the samples and with <10 reads was done. Finally, ten random milk and plasma subsamples of 0.2M miRNA reads each (the minimum in plasma samples) were obtained and analysed. P values of each of the 10 differential analysis were combined using simes test [36], from mppa v.1.0 (eprint arXiv:1408.3845) in R (3.3.2).

## Results

### Study population and sample collection

Fifteen healthy volunteer mothers were enrolled in the study during 24-72h post-delivery. Main characteristics of the mothers are found in Table 1. Mean age of the participants was 32.9 years. Forty percent of them were primiparous and only one of them smoked during pregnancy. One third of the participants were born in Spain, one third in Asia and the rest were from Morocco and South America. Plasma samples were collected for all 15 women, while milk was available for 10 of them.

**Table 1:** Main characteristics of study participants.

Variable	Summary
Age (years); mean (range)	32.93 (23–40)
Parity; n (%)	
Primigravidae	6 (40%)
Multigravidae	9 (60%)
Region of origin; n (%)	
Spain	5 (33.3%)
Asia	5 (33.3%)
Morocco	2 (13.3%)
South America	3 (20.1%)
Tobacco smoking during pregnancy; n (%)	
Yes	1 (6.7%)
No	14 (93.3%)

### RNA extraction and quality control

The average amount of RNA extracted from plasma and milk samples was 81.14 ng/mL (standard deviation, SD 40.92) and 3,984.85 ng/mL (SD 3,766.97), respectively (S1 Table). In the Bioanalyzer plot, all biofluids showed a peak between 25 to 200 nucleotides which corresponded to the expected size for small RNAs (S2 Fig). No peaks were detected at 18S and 28S in plasma, whereas a peak was observed in half of the milk samples, suggesting potential contamination with cellular RNA.

### Quality control

The mean number of reads obtained for each biofluid was similar: 13.96 million (M) in plasma and 13.87 M in milk. However, the average proportion of good quality reads was 89.58% in milk, and only 40.01% in plasma (Table 2 and S2 Table). Three point ninety seven percent and 35.72% of the raw reads mapped to miRNAs in plasma and milk, respectively. Only samples with >2M of good quality reads of which >0.2 M mapped to miRNAs were considered for further analysis (10 milk and 5 plasma samples).

**Table 2:** Quality control and mapped reads to miRNAs [mean and (SD)], by biofluid.

		Plasma	Milk
		(N = 15)	(N = 10)
Total reads; millions (SD)		13.96 (16.71)	13.87 (8.23)
Quality filtered; millions (SD)		9.82e-3 (9.75e-3)	6.33e-3 (1.98e-3)
Complexity filtered; millions (SD)		1.40e-4 (1.32e-4)	1.39e-4 (7.26e-5)
Size filtered; millions (SD)		9.92 (11.40)	1.36 (0.86)
Good quality reads^a^			
	Millions (SD)	4.04 (6.16)	12.52 (7.74)
	% raw reads (SD)	40.01 (23.05)	89.58 (5.42)
miRNA			
	Millions (SD)	0.27 (0.44)	5.42 (4.88)
	% raw reads (SD)	3.97 (4.36)	35.72 (12.40)
% QCed reads (SD)		8.92 (6.85)	40.02 (13.43)
N samples tested^b^		5	10

^a^ Reads after filtering low quality, low complexity and size <18 nt.

^b^ Number of samples tested in the downstream differential analysis of miRNAs, isomiRs and small RNA clusters (>0.2M reads in miRNAs).

### miRNAs

**Fig 1:**
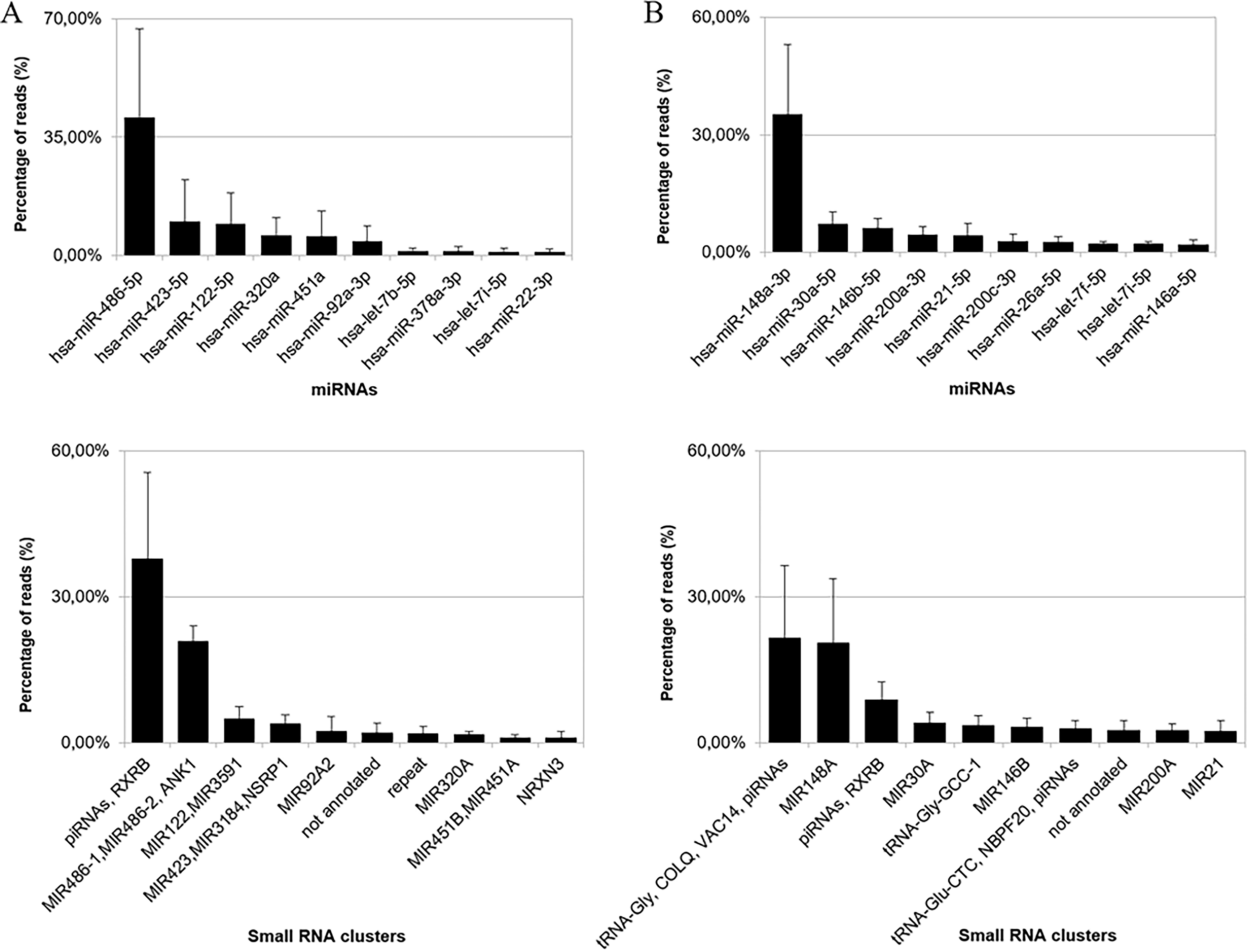
Relative abundance of miRNA and small RNA clusters. (A, C) Plasma, (B, D) milk. The x-axis represents miRNAs (A, B) or small RNA clusters (C, D) ordered according to their expression level and the y-axis represents the abundance as percentage of reads (%) (average of all samples). Error bar shows SD.

**Fig 2:**
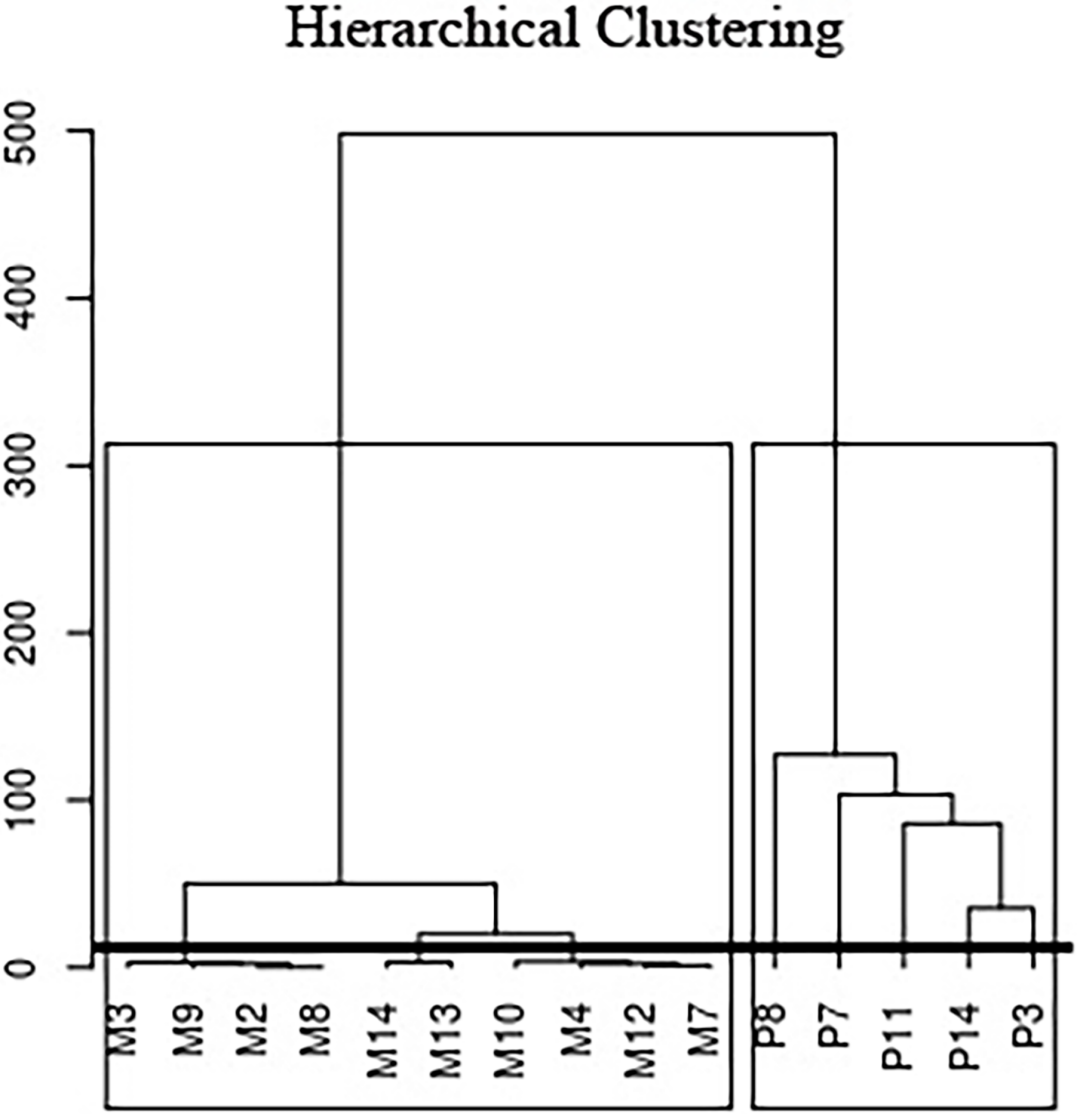
Dendogram of samples according to their normalized miRNA levels (10 milk samples (M) and 5 plasma samples (P)). Samples are classified by biofluid and not by individual as indicated by the squares.

Total number of miRNAs detected with >1 count were 1,182: 1,002 in milk [456 with a mean abundance of >10 reads per million (RPM)] and 824 in plasma [271 with a mean abundance of >10 RPM]. Ten miRNAs comprised >70% of the expression in each biofluid (Fig 1, S3 Table). miRNA complexity was quite heterogeneous among samples, even among samples from the same biofluid, and tended to be higher in milk than in plasma (S3 Fig). Samples clustered by biofluid and not by subject (Fig 2). Data did not cluster by technical factors (time to storage, RNA extraction date, library preparation date, sequencing run and lane) or demographic/biological variables (age, region of origin, and parity) (S4 Fig).

At 5% FDR, 308 miRNAs showed different levels between biofluids [126 (40.91%) milk > plasma] (Table 3 and S4 Table). Among the 112 miRNAs found in >80% of the samples and with >10 counts, 87 of them showed statistically significant differences between biofluids (66 milk > plasma). Seventy-four out of the 87 miRNAs overlapped with the ones detected in the main analysis (S7 Fig).

**Table 3:** Differential levels of miRNAs by biofluid—top 10 ordered by p value.

**A.** Top ten higher in plasma				
**miRNA**	**baseMean**	**log2FC**	**p value**	**adjusted p value**
hsa-miR-451a	26988.57	11.57	5.09e-62	3.59e-59
hsa-miR-122-5p	117899.75	13.14	9.14e-47	3.23e-44
hsa-miR-486-5p	531306.29	14.15	1.26e-42	2.96e-40
hsa-miR-4508	790.10	10.21	5.36e-38	9.47e-36
hsa-miR-134-5p	500.38	9.47	6.17e-24	8.71e-22
hsa-miR-363-3p	451.88	6.03	2.53e-21	2.98e-19
hsa-miR-584-5p	319.49	7.77	8.87e-21	8.95e-19
hsa-miR-150-5p	815.24	6.25	2.00e-20	1.66e-18
hsa-miR-370-3p	317.26	9.52	2.11e-20	1.66e-18
hsa-miR-127-3p	670.08	7.22	8.02e-20	5.66e-18
**B.** Top ten higher in milk				
**miRNA**	**baseMean**	**log2FC**	**p value**	**adjusted p value**
hsa-miR-200b-3p	22349.79	-7.74	2.60e-19	1.67e-17
hsa-miR-30a-5p	90191.79	-5.59	1.34e-18	7.88e-17
hsa-miR-200a-3p	55145.39	-7.13	4.16e-18	2.26e-16
hsa-miR-200c-3p	31313.85	-7.34	7.23e-18	3.64e-16
hsa-miR-146b-5p	73258.87	-5.00	2.70e-17	1.27e-15
hsa-miR-200a-5p	2472.99	-8.19	4.87e-15	2.02e-13
hsa-miR-30b-5p	2335.32	-6.24	9.53e-14	3.54e-12
hsa-miR-141-3p	9204.94	-7.08	1.48e-13	5.24e-12
hsa-miR-193b-3p	797.36	-6.97	1.84e-13	6.18e-12
hsa-miR-30a-3p	5492.94	-5.51	2.22e-12	6.27e-11

Analysis was done with DESEq2. baseMean is the mean of normalized counts for all samples. log2FC is the log2 fold change between plasma and milk samples. Adjusted p value by the Benjamini-Hochberg method.

To further confirm the results given the different sequencing depth of each biofluid, 10 random subsamples of 0.2 M miRNA reads were obtained for each plasma and milk sample. The total number of unique miRNA in the 10 milk subsamples was 534 (115 common to all milk samples) and in the 10 plasma subsamples was 715 (100 common to all plasma samples). Samples were again classified by biofluid (S8 Fig). Two-hundred and fourteen miRNAs exhibited different levels depending on the biofluid [72 (33.64%) milk > plasma]. One hundred and eighty six overlapped with the results of the main analysis (S7 Fig, S4 Table).

### isomiRs

Total number of isomiRs with >1 count was 10,541 in milk (1,798 with mean abundance of >10 RPM) and 4,760 in plasma (1,376 with mean abundance of >10 RPM) (S5 Table). Some miRNAs bear more than one modification type. In plasma, 3’ additions, 3’ trimming and 5’ trimming modifications represented 31.77%, 48.20%, and 20.03% of all editions, respectively. In milk, distributions were rather similar (3’ additions: 34.04%, 3’ trimmings: 44.91%, and 5’ trimmings: 21.05%). Table 4 describes the distribution and abundance by biofluid of different types of isomiRs, as combinations of 1, 2 or 3 modification types. The most common combinations in terms of number of isomiRs were: 3’ addition + 3’ trimming (27.98% in plasma and 34.39% in milk) and 3’ trimmings (27.79% in plasma and 18.35% in milk). Regarding relative abundance, isomiRs with 3’ trimming changes accounted for around 70% of total reads in both biofluids. They were followed by isomiRs with 3’ additions, in plasma, and by isomiRs with a combination of 3’ trimmings + 3’ additions, in milk.

**Table 4:** Number and relative abundance of different types of isomiRs, as combinations of different editions, in milk and plasma.

	Number of isomiRs^a^				Relative abundance (%)^b^			
	Plasma (N = 4760)		Milk (N = 10541)		Plasma		Milk	
isomiR with different modifications:	N	%	N	%	Mean	SD	Mean	SD
3' addition	549	11.53%	987	9.36%	21.75%	7.86	6.41%	1.24
3' addition + 3' trimming	1332	27.98%	3625	34.39%	8.11%	2.42	19.33%	6.98
3' addition + 3' trimming + 5' trimming	406	8.53%	1358	12.88%	0.35%	0.20	0.95%	0.35
3' addition + 5' trimming	181	3.80%	489	4.64%	0.24%	0.07	0.26%	0.11
3' trimming	1323	27.79%	1934	18.35%	66.89%	8.47	70.66%	8.46
3' trimming + 5' trimming	683	14.35%	1604	15.22%	1.76%	0.99	1.54%	0.15
5' trimming	286	6.01%	544	5.16%	0.90%	0.41	0.85%	0.18

At a 5% FDR, 1,790 isomiRs (variants of 298 different miRNAs) showed different levels between biofluids [1,266 (70.73%) milk > plasma] (S6 Table).

### Small RNA clusters

In total, one thousand and fifty three small RNA clusters were identified in the 10 milk and 5 plasma samples. One thousand and three of them were found in milk with >1 count (930 with mean abundance of >10 RPM), and 819 in plasma (648 with mean abundance of >10 RPM). Only for descriptive purposes, clusters with multiple annotations were classified to one single RNA class following this prioritization: miRNA, rRNA, tRNA, snoRNA, small nuclear RNA (snRNA), vault RNAs (VTRNA), long non-coding RNA (lncRNA), piRNA, gene, and repeats. The number and proportion of clusters in each RNA class by biofluid can be found in Table 5. In both biofluids, more than 30% of the clusters identified were annotated to genes, and around 20% to miRNAs. Milk samples showed a higher proportion of reads mapping to miRNAs (46.76%) and tRNAs (30.91%), whereas plasma had a higher proportion of reads in clusters represented by piRNAs (32.03%), genes (6.25%), and repeats (40.57%) (Table 5). miRNA clusters in plasma corresponded to 18.20% of the total number of reads. Around 6% and 3% of the reads remained not annotated in plasma and milk, respectively.

**Table 5:** Number of clusters and abundance in milk and plasma.

	Number of clusters				Relative abundance (%)^a^			
	Plasma (N = 819)		Milk (N = 1,003)		Plasma		Milk	
Cluster class	N	%	N	%	Mean	SD	Mean	SD
miRNA	181	22.10	191	19.043	18.20	11.88	46.76	16.41
rRNA	5	0.61	6	0.60	0.01	0.01	0.01	0.00
tRNA	78	9.52	85	8.48	1.00	0.23	30.91	15.49
snoRNA	33	4.03	34	3.39	0.07	0.04	0.24	0.14
snRNA	3	0.37	4	0.40	0.03	0.03	0.01	0.01
VTRNA	3	0.37	3	0.30	0.01	0.01	0.01	0.01
lncRNA	14	1.71	19	1.89	0.36	0.20	0.03	0.01
piRNA	9	1.10	12	1.20	32.03	5.86	8.42	2.94
gene	259	31.62	421	41.97	6.25	2.80	0.93	0.32
repeat	108	13.19	97	9.67	40.57	36.66	10.02	3.44
not annotated	126	15.38	131	13.06	6.44	5.38	2.66	1.75

^a^ Average of relative abundance of all samples (number of reads of each cluster class in a particular sample divided by total number of reads in that sample).

Similarly to the miRNA analysis, the 10 most abundant clusters represented >75% of the reads in each biofluid (Fig 1, S7 Table). In milk, a tRNA cluster (*tRNA-Gly-GCC*, *tRNA-Gly-CCC*2, *COLQ*, *VAC14*, and several piRNAs) accounted for 21.67% of the reads, followed by *MIR148A* with 20.69%. In plasma, 37.94% were from a cluster that mapped to several piRNAs and *RXRB* gene. The second most abundant cluster, with 21.04% of the reads, included *MIR486-1*, *MIR486-2*, and *ANK1*gene. Abundance profile along precursor of these four clusters is shown in S9 Fig.

The levels of 774 clusters were statistically significant different between plasma and milk [291 (37.60%) milk > plasma] (Table 6 and S8 Table). Besides differently detected miRNAs, clusters of other RNA classes were identified. They included clusters mapped to genes (*TSHZ2*, plasma>milk), tRNAs (*tRNA-Trp-CCA-2-1*, milk > plasma), VTRNs (*VTRNA2-1*, plasma > milk), snoRNAs (*SNORD95*, plasma > milk), or lncRNAs (*HOTAIR*, plasma > milk). Their profiles can be seen in S10 Fig. When considering isomiRs or small RNA clusters instead of miRNAs, samples also grouped by biofluid (S5 and S6 Figs).

**Table 6:** Differential levels of small RNA clusters by biofluid—top 10 ordered by p value.

**A.** Top ten higher in plasma						
**Cluster id**	**Cluster class**	**annotation**	**baseMean**	**log2FC**	**p value**	**adjusted p value**
141	miRNA	*MIR451B*, *MIR451A*^a^	27640.81	11.76	1.44e-48	1.53e-45
695	miRNA	*MIR122*^a^, *MIR3591*	114740.42	13.24	3.05e-36	1.61e-33
815	miRNA	*MIR486-1*^a^, *MIR486-2*^a^, *ANK1*	581529.14	14.32	6.79e-35	2.39e-32
114	miRNA	*MIR150*^a^	878.09	6.47	3.93e-18	1.04e-15
362	gene	*TSHZ2*, *repeat*	1745.85	8.87	1.41e-17	2.99e-15
671	miRNA	*MIR127*^a^, *RTL1*	607.44	7.54	1.43e-16	2.51e-14
390	miRNA	*MIR363*^a^	434.99	6.20	4.46e-15	5.89e-13
30	repeat	*Repeat*	4220.07	8.31	7.15e-15	8.40e-13
314	gene	*ARHGEF38*, *repeat*	3069.65	8.22	9.92e-15	1.05e-12
361	not annotated	*not annotated*	1429.83	7.93	1.39e-14	1.23e-12
**B.** Top ten higher in milk						
**Cluster id**	**Cluster class**	**annotation**	**baseMean**	**log2FC**	**p value**	**adjusted p value**
128	miRNA	*MIR200B*^a^	23506.88	-7.91	8.64e-16	1.30e-13
127	miRNA	*MIR200A*^a^	59945.49	-7.31	1.22e-14	1.17e-12
130	miRNA	*MIR200C*^a^, *LOC105369635*	31561.40	-7.42	2.74e-14	2.23e-12
963	miRNA	*MIR30A*^a^	98503.77	-5.61	1.19e-13	7.88e-12
943	miRNA	*MIR146B*^a^	76866.79	-5.01	1.40e-12	6.18e-11
659	miRNA	*MIR335*, *MeST*	2261.74	-6.84	2.24e-12	9.47e-11
825	tRNA	*tRNA-Trp-CCA-2-1*	1432.83	-6.70	1.64e-11	5.98e-10
794	tRNA	*tRNA-Asp-GTC-1-1*, *LOC643770*	2532.81	-8.52	5.14e-11	1.65e-09
131	miRNA	*MIR141*^a^, *LOC105369635*	9289.10	-7.18	7.28e-11	2.20e-09
849	tRNA	*tRNA-Val-TAC-1*	2848.07	-5.87	1.58e-10	4.19e-09

Analysis was done with DESEq2. baseMean is the mean of normalized counts for all samples. log2FC is the log2 fold change between plasma and milk samples. Adjusted p value by the Benjamini-Hochberg method

^a^ Detected among the top 10 findings in the miRNA analysis.

## Discussion

In this study, we identified several species of small RNAs, including miRNAs, piRNAs, tRNAs, snRNAs and snoRNAs in both plasma and breast milk. Their profiles were biofluid specific. As the identification of circulating small RNAs depends on the quality of the biological material, the quantification method and the bioinformatic pipeline, we discuss bellow the technical aspects followed during this study that can potentially affect the results obtained.

### RNA extraction and sequencing

The RNA extraction and sequencing yielded data of enough quality for all milk samples and 5 out of the 15 plasmas. Forty % of reads in plasma and 89.58% in milk passed the quality control step, with only 3.97% and 35.72% mapping to miRNAs, respectively. RNA input for plasma and milk small RNA libraries were 150 ng and 500 ng, respectively, which respond to the different RNA quantity obtained for each biofluid. RNA yield is thus a key factor for the quality of sequencing data.

In plasma, the lack of 18S and 28S peaks in the RNA patterns obtained with the Bioanalyzer suggested the absence of RNA cell contamination. Indeed, plasma samples were centrifuged twice in <3.5 h after blood collection and the spectrophotometer haemolysis values were below 0.2, except for one sample. Given the low RNA yield obtained per mL of plasma (around 40 ng/mL), the addition of carriers during RNA extraction would be recommended [37].

In contrast, milk samples presented some signal at 18S and 28S, independently of whether samples were centrifuged one or two times before storing. Milk samples also showed systematically higher RNA levels by mL of biofluid than plasma samples (around 50-times higher levels). Given the higher amount of RNA obtained in milk and the presence of ribosomal RNA, we suspect contamination with the cellular fraction during skim milk sample separation. However, other studies reported similar RNA yields for skim milk (around 4000 ng/mL) as ours, and higher amounts in the milk fat and cellular fractions [38]. Contamination with cellular RNA is one of the main issues in the use of circulating free small RNAs as biomarkers of disease [39]. Therefore, all the findings shown here, as well as in other publications, should be interpreted considering this potential limitation.

### Small RNA bioinformatic analysis

Currently, the analysis of miRNA sequencing data is quite straight forward. In contrast, differences of one or few nucleotides in isomiRs with respect to reference miRNAs and frequent multicopy non-miRNA small RNAs in the genome impose several limitations to their bioinformatics analysis. To quantify small RNAs, we used SeqCluster, a tool that groups RNAs in clusters based on their sequence similarity. RNA clusters are defined as unique transcriptions units with potentially the same molecular function given their similar sequence, regardless of their genomic location. For instance, tRNAs which are present in different copies in the genome are clustered together. The reference databases and approach used in this study differ from the pipeline followed in two of the most recent publications on circulating small RNAs: Freedman et al. 2016 (ExceRpt, based on sRNABench) [19] and Yeri et al. 2017 (sRNABench) [21]. In sRNABench, reads are first mapped to miRNA, and remaining reads are mapped to other small RNAs using ENSEMBL 75. For isomiRs characterization, 3’ additions and 3’ and 5’ trimming editions were considered in the analysis, as there are strong evidences of their processing [40–42]. In contrast, isomiRs with substitutions were discarded due to difficulty of mapping them unambiguously.

### Small RNAs in plasma

Ten out of the 824 miRNAs detected in plasma represented >70% of total number of reads mapping to miRNAs. Other authors have also reported few miRNAs accounting for around 50% of the total number of sequencing reads [19, 43]. In agreement with Yeri et al. [21] hsa-miR-486-5p was the most abundant miRNA in plasma (representing approximately 40% of the miRNA reads). A bias towards this miRNA in samples prepared with the Illumina kit has been reported in plasma-derived exosomes [20]. Ten and 8 of the 10 top miRNAs identified in this study were detected among the top 50 positions in Freedman et al. [19] and in Yeri et al. [21], respectively. The most abundant miRNA in Freedman et al was hsa-miR-451a, while in our sample set this miRNA was found in the 5^th^ position. According to public data deposited in the miRmine database [44], hsa-miR-486-5p and hsa-miR-451a are the two most abundant miRNAs in plasma, and are produced by red blood cells [39]. Hsa-miR-122, which is highly abundant in plasma, is mostly expressed in liver and enters easily into circulation, pointing towards its potential as biomarker for several liver pathologies [45].

In agreement with Freedman et al. [19] and Yeri et al. [21] we also found piRNAs, tRNAs, snoRNAs, snRNAs, VTRNAs and lncRNAs in plasma. The abundance of miRNAs, tRNAs, snoRNAs, snRNAs, and VTRNAs was in a similar range among previous studies. In contrast, there were strong discrepancies in the piRNA levels. Interestingly, a piRNA cluster mapping to several piRNAs and *RXRB* gene accounted for 37.94% of the reads in the plasma samples analysed in this study. After visual inspection of the genomic region, we realized that this cluster also overlaps with *Y RNA* genes (*RNAY4/RNAY4P10*). Indeed, Yeri et al. (2017) described a high proportion of sequences mapping to *Y RNA* in plasma (>60%) and a much lower abundance of piRNAs [21] *Y RNAs* participate in chromosomal DNA replication and are involved in RNA stability and response to stress [46]. Circulating *Y RNAs* of 25–33 nt have been found in blood, within vesicles or as cell-free ribonucleoprotein (RNP) complexes [47–49]. Their function in biofluids, if any, is unknown and it cannot be discarded whether they are degradation products.

piRNAs were thought to be specific to germ cells, where they play essential roles in fertility and regeneration [50], but they have been described in plasma [19, 21], urine, and saliva [21] and in healthy and cancer somatic cells [51, 52]. In particular, Keam et al showed that Hiwi2 protein, one of the three human Piwi proteins, was ubiquitously expressed in somatic cells. The sequencing of the RNA co-immunoprecipitated with Hiwi2 protein identified a wide range of small RNA sequences of 18 to 34 nt that mapped to processed tRNA fragments, known piRNAs and coding genes, among others. The authors suggest that, in somatic cells, the Piwi-piRNA pathway participates in the regulation of protein translation [51]. Of note, the way how piRNAs are identified, through sequencing of Piwi bound RNAs, might introduce some noise in the piRNA sequence pool in reference databases.

Differences in small RNA abundance with previous publications [19, 21] can be explained by biological and technical factors. The quantification of small RNAs was performed using different NGS platforms, for which some bias to specific small RNAs have been described before [20]. Moreover, these studies used different bioinformatics pipelines. The small sample size of our dataset (5 plasma samples) in combination with the low and highly heterogeneous expression pattern of small RNAs in plasma with only a few of them detected in all samples might have biased our findings. Finally, our study included a highly specific population of post-partum mothers, while Freedman et al. and Yeri et al. enrolled participants from the general population and male athletes, respectively.

### Small RNAs in milk

Several studies have analysed miRNAs in different milk fractions (whole milk [53], skim milk [38, 54, 55], milk fat [38, 53, 55], or milk extracellular vesicles [56–58]), in different lactation points, and using different methods (NGS [53, 56, 58], qPCR [55, 57], or microarrays [54]). Long non-coding RNAs in milk microvesicles have also been reported [57].

In the present study focusing on skim milk, 10 miRNAs accounted for >70% of the reads mapped to miRNAs. Hsa-miR-148a-3p represented around 30% of the reads. This miRNA was previously reported to be an abundant miRNA in human milk fat [53] and milk exosomes/microvesicles [56, 58]. Of notice, this miRNA was only found in studies using NGS, but not those applying qPCR or microarrays. Hsa-miR-146b, hsa-miR-200c and hsa-30a-5p, among top miRNAs expressed in our sample set, were also reported to be highly abundant in milk fat, skim milk or milk exosomes/microvesicles [53, 55, 56, 58]. Moreover, some of them were also abundant in the milk cellular fractions [55]. A direct comparison of this study versus previous publications is subjected to the differences in starting biological material, lactation period, and quantification methods.

Besides miRNAs, we detected other small RNAs, being tRNAs one of the most abundant class. tRNAs have been found before in several biofluids [19, 21], including milk exosomes [56, 58]. tRNA fragments seem to have distinct expression patterns and regulatory functions [59]. As far as we know, this is the first study describing the presence of piRNAs, snRNAs, snoRNAs and VTRNAs in milk. Whether breast milk tRNAs and other small RNAs can reach child circulation and modulate child physiology is of great interest and requires further investigation.

### Differential detection of RNAs in both biofluids

As expected, we observed a clear separation between biofluids in terms of their small RNA profile [21, 60]. Previous studies have shown that the miRNA profile observed in each biofluid recapitulates the miRNA profile of the main cell types releasing small RNAs in those biofluids (blood cells in plasma [39] and mammary epithelial cells in milk [55]).

Besides miRNAs, several small RNA clusters showed a different expression pattern between biofluids. As an example, levels of *VTRNA2-1* were higher in plasma than in milk. *VTRNA2-1* transcript is a putative tumour suppressor and modulator of innate immunity, which responds to environmental stressors during development through loss of imprinting [61]. *HOTAIR*, also detected at higher levels in plasma, is a long non-coding RNA that represses transcription of *HOXD* genes in trans. *HOTAIR* has been implicated in cancer [62]. We detected >10,000 isomiRs which beard one or several editions (3’ addition, 3’ trimming or 5’ trimming): 4,760 in plasma and 10,541 in milk, processed from 707 and 875 miRNAs, respectively. Differentially isomiRs found in milk and plasma matched with the differentially expressed miRNAs detected in these two biofluids. Some studies suggest that specific isoforms of the same miRNA are the ones that have an effect on disease [63]. Further investigation is needed to corroborate whether isomiRs affected by specific diseases can be found in circulation, however our study shows a myriad of isomiRs which could be potentially used as biomarkers.

Although sequencing depth of milk and plasma samples was similar, the proportion of good quality reads mapping to miRNA was much higher in milk than in plasma. This could bias the results of the differential analysis (i.e. some miRNAs expressed at mid or low levels in milk might not have been detected in plasma due to the lower sequencing depth). DESeq2 normalizes using the geometric median of the ratio between the gene of each sample and the average among samples, but when there are important differences in library size, normalization methods might not to be sufficient [64]. To confirm the biofluid-specific findings, we applied a subsampling approach in the miRNAs analysis. This approach consists in a random selection of the same read number in all the samples of the study. To control for a potential selection bias, we performed ten random subsamplings. Approximately 60% (186 out of 308) of the miRNAs detected in the main differential analysis were confirmed in the subsampling analysis. miRNAs only detected in the main analysis and not in the subsampling analysis could be false positives resulting from the different depth of sequencing between biofluids.

### Summary

Plasma and milk samples showed a completely different small RNA profile. Several types of small RNA species were detected in both biofluids, including miRNAs, tRNAs, snRNAs, snoRNAs, lncRNAs, and piRNAs. The presence of different species of small RNAs in biofluids, besides miRNAs, opens the door to explore them as potential biomarkers of disease or as mediators of lactation health effects.

## Supporting information

S1 Fig Bioinformatic and statistical analysis pipeline. (TIF)

S2 Fig Bioanalyzer RNA pattern for representative samples. Plasma (A) and milk (B–low cell contamination, C–potential cell contamination) samples.
(TIF)

S3 Fig miRNA complexity per sample. miRNA complexity is defined as the number of miRNA genes that are observed as a function of the number of miRNA reads. The x-axis represents the number of reads (the total number is indicated on the right). The gradient colour of the bar from white to black shows the incremental detection of distinct miRNA genes as more of the sequenced reads are considered.
(TIF)

S4 Fig Principal Component Analysis of normalized miRNA count data (10 milk and 5 plasma samples). A) Heat-map of the association between the first five principal components and the technical and biological variables. SAMPLE_TYPE and STARTING_RNA (RNA input for library preparation, which is related to SAMPLE_TYPE) are strongly associated with the first principal component having both a p-value of association of 8.44e-08. B) Accumulated variance explained by the first 10 principal components. In green, the bars corresponding to principal component one and two are highlighted. The sea-green line indicates the accumulated explained variance in each principal component. The dark-red dashed lines indicate the principal component which accumulated explained variance overload 90%, which corresponds to the ninth principal component with an explained variance of 90.82%. C) Scatter plot of the samples located on the two first principal components. Samples cluster in two groups corresponding to milk (red) and plasma (blue).
(TIF)

S5 Fig Principal Component Analysis of normalized isomiRs count data (10 milk and 5 plasma samples). A) Heat-map of the association between the first five principal components and the technical and biological variables. SAMPLE_TYPE and STARTING_RNA (RNA input for library preparation, which is related to SAMPLE_TYPE) are strongly associated with the first principal component having both a p-value of association of 0.002. B) Accumulated variance explained by the first 10 principal components. In green, the bars corresponding to principal component one and two are highlighted. The sea-green line indicates the accumulated explained variance in each principal component. The dark-red dashed lines indicate the principal component which accumulated explained variance overload 90%, which corresponds to the tenth principal component with an explained variance of 92.86%. C) Scatter plot of the samples located on the two first principal components. Samples cluster in two groups corresponds to milk (red) and plasma (blue).
(TIF)

S6 Fig Principal Component Analysis of normalized small RNA clusters count data (10 milk and 5 plasma samples). A) Heat-map of the association between the first five principal components and the technical and biological variables. SAMPLE_TYPE and STARTING_RNA (RNA input for library preparation, which is related to SAMPLE_TYPE) are highly associated with the first principal component having both a p-value of association of 1.52e-07. B) Accumulated variance explained by the first 10 principal components. In green, the bars corresponding to principal component one and two are highlighted. The sea-green line indicates the accumulated explained variance in each principal component. The dark-red dashed lines indicate the principal component which accumulated explained variance overload 90%, which corresponds to the seventh principal component with an explained variance of 91.04%. C) Scatter plot of the samples located on the first two principal components. Samples cluster in two groups corresponding to milk (red) and plasma (blue).
(TIF)

S7 Fig Venn diagrams of overlapped differentially expressed miRNAs according to different filtering strategies. (TIF)

S8 Fig Dendogram of miRNA expression levels in milk and plasma subsamples. For each sample (10 milk and 5 plasmas) 10 random subsamples of 0.2M reads of miRNA were obtained. Samples classify by biofluid and by sample of origin. Coloured branches represent 10 subsamples of the same sample of origin and bottom boxes the biofluid.
(TIF)

S9 Fig Abundance profile along precursor for main RNA clusters detected in milk and plasma. Y-axis represents the abundance profile, and x-axis the positions in the precursor. A) Milk: *MIR148A*, B) Milk: tRNA-Gly-GCC and tRNA-Gly-CCC; C) Plasma: Several piRNAs and *RXRB* gene; D) Plasma: *MIR486-1*, *MIR486-2*, and *ANK1* gene. In orange milk samples, and in blue plasma samples.
(TIF)

S10 Fig Abundance profile along precursor for a set of representative small RNA clusters differentially expressed in milk and plasma. Y-axis represents the abundance profile, and x-axis the positions in the precursor. A) HOTAIR, higher levels in plasma, B) SNORD95, higher levels in plasma; C) tRNA-Trp-CCA-2-1, higher levels in milk; D) VTRNA2-1, higher levels in plasma. In orange milk samples, and in blue plasma samples.
(TIF)

S1 Table RNA concentration (ng/ml) and purity, by sample. (XLSX)

S2 Table Quality control and mapped reads [mean and (SD)] in different species of small RNAs, by sample. (XLSX)

S3 Table Relative abundance of miRNAs in plasma and milk. (XLSX)

S4 Table Differential detection of miRNAs by biofluid—ordered by p value. (XLSX)

S5 Table Relative abundance of isomiRs in plasma and milk. (XLS)

S6 Table Differential detection of isomiRs by biofluid—ordered by p value. (XLS)

S7 Table Relative abundance of small RNA clusters in plasma and milk. (XLSX)

S8 Table Differential detection of RNA clusters by biofluid—ordered by p value. (XLSX)
