# Supplementary figures and images for: Circulating miRNAs, isomiRs and small RNA clusters in human plasma and breast milk
Source: PLoS One. 2018 Mar 5;13(3):e0193527. doi: 10.1371/journal.pone.0193527 (PMC5837101; doi:10.1371/journal.pone.0193527)

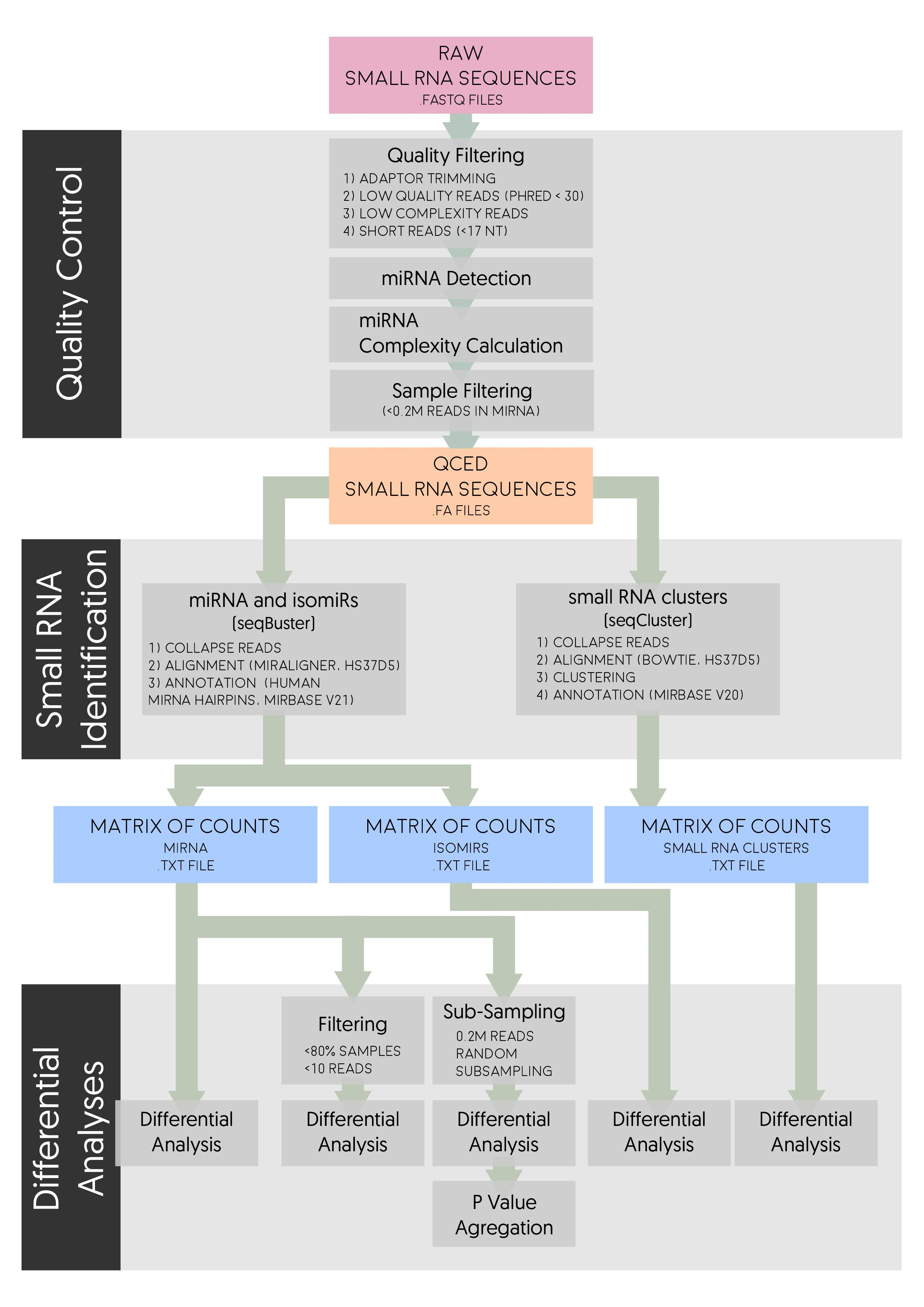

Supplement: S1 Fig — (TIF) [file pone.0193527.s001.tif]

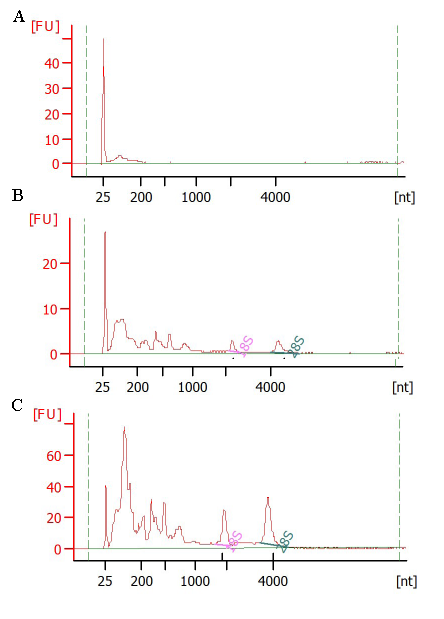

Supplement: S2 Fig — Plasma (A) and milk (B–low cell contamination, C–potential cell contamination) samples. (TIF) [file pone.0193527.s002.tif]

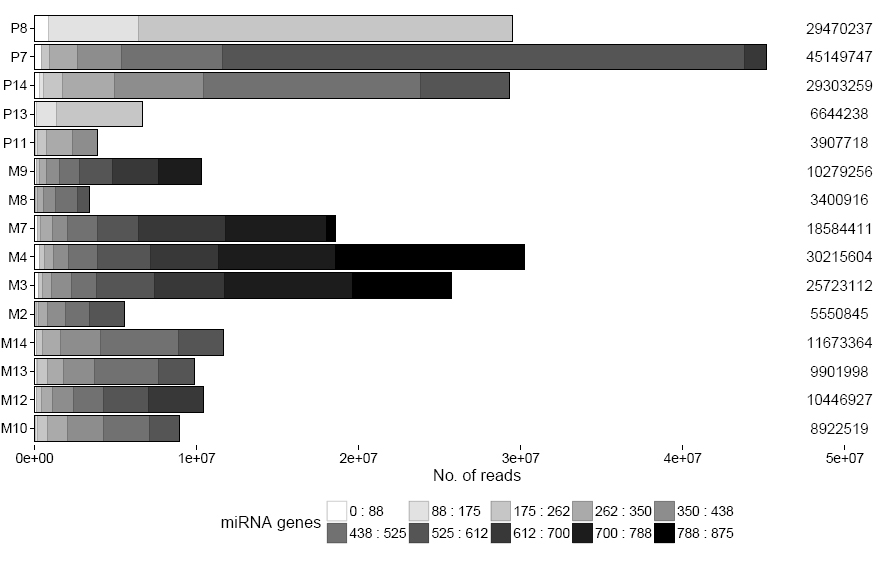

Supplement: S3 Fig — miRNA complexity is defined as the number of miRNA genes that are observed as a function of the number of miRNA reads. The x-axis represents the number of reads (the total number is indicated on the right). The gradient colour of the bar from white to black shows the incremental detection of distinct miRNA genes as more of the sequenced reads are considered. (TIF) [file pone.0193527.s003.tif]

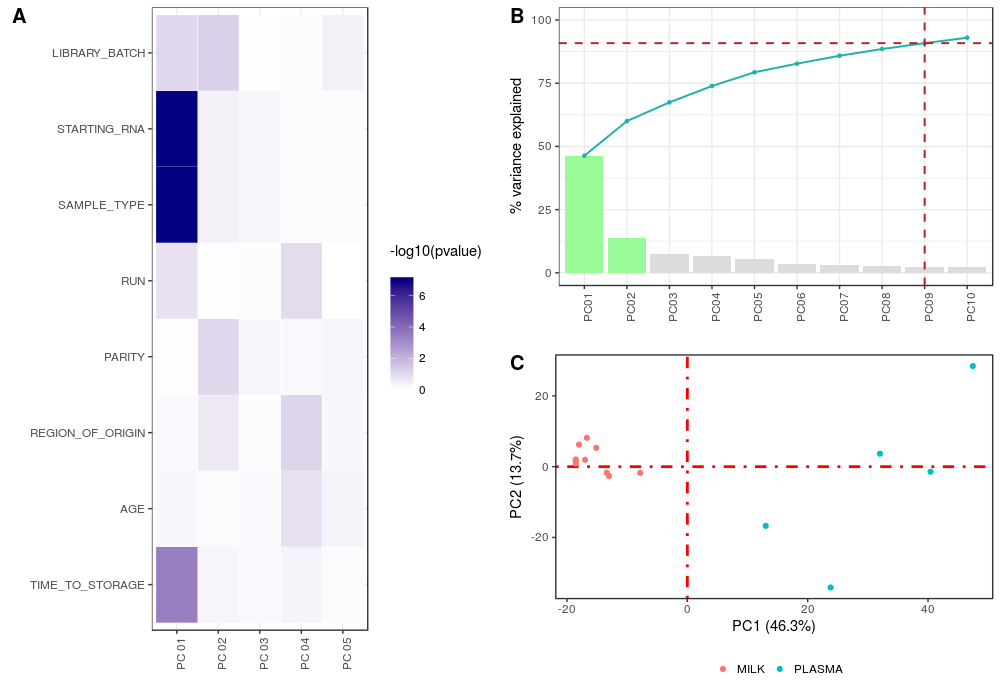

Supplement: S4 Fig — A) Heat-map of the association between the first five principal components and the technical and biological variables. SAMPLE_TYPE and STARTING_RNA (RNA input for library preparation, which is related to SAMPLE_TYPE) are strongly associated with the first principal component having both a p-value of association of 8.44e-08. B) Accumulated variance explained by the first 10 principal components. In green, the bars corresponding to principal component one and two are highlighted. The sea-green line indicates the accumulated explained variance in each principal component. The dark-red dashed lines indicate the principal component which accumulated explained variance overload 90%, which corresponds to the ninth principal component with an explained variance of 90.82%. C) Scatter plot of the samples located on the two first principal components. Samples cluster in two groups corresponding to milk (red) and plasma (blue). (TIF) [file pone.0193527.s004.tif]

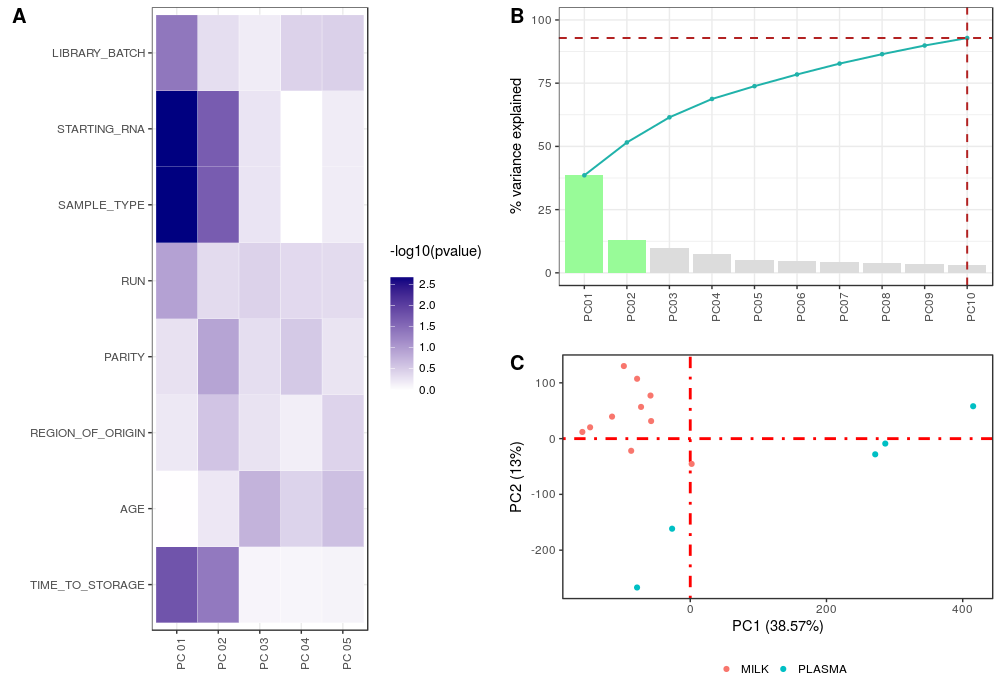

Supplement: S5 Fig — A) Heat-map of the association between the first five principal components and the technical and biological variables. SAMPLE_TYPE and STARTING_RNA (RNA input for library preparation, which is related to SAMPLE_TYPE) are strongly associated with the first principal component having both a p-value of association of 0.002. B) Accumulated variance explained by the first 10 principal components. In green, the bars corresponding to principal component one and two are highlighted. The sea-green line indicates the accumulated explained variance in each principal component. The dark-red dashed lines indicate the principal component which accumulated explained variance overload 90%, which corresponds to the tenth principal component with an explained variance of 92.86%. C) Scatter plot of the samples located on the two first principal components. Samples cluster in two groups corresponds to milk (red) and plasma (blue). (TIF) [file pone.0193527.s005.tif]

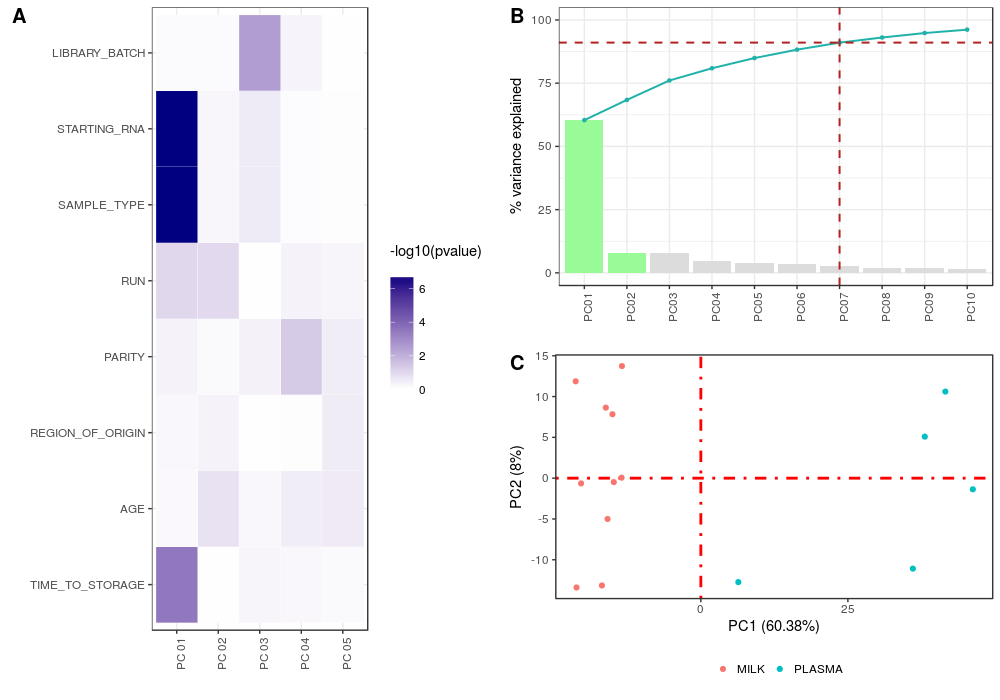

Supplement: S6 Fig — A) Heat-map of the association between the first five principal components and the technical and biological variables. SAMPLE_TYPE and STARTING_RNA (RNA input for library preparation, which is related to SAMPLE_TYPE) are highly associated with the first principal component having both a p-value of association of 1.52e-07. B) Accumulated variance explained by the first 10 principal components. In green, the bars corresponding to principal component one and two are highlighted. The sea-green line indicates the accumulated explained variance in each principal component. The dark-red dashed lines indicate the principal component which accumulated explained variance overload 90%, which corresponds to the seventh principal component with an explained variance of 91.04%. C) Scatter plot of the samples located on the first two principal components. Samples cluster in two groups corresponding to milk (red) and plasma (blue). (TIF) [file pone.0193527.s006.tif]

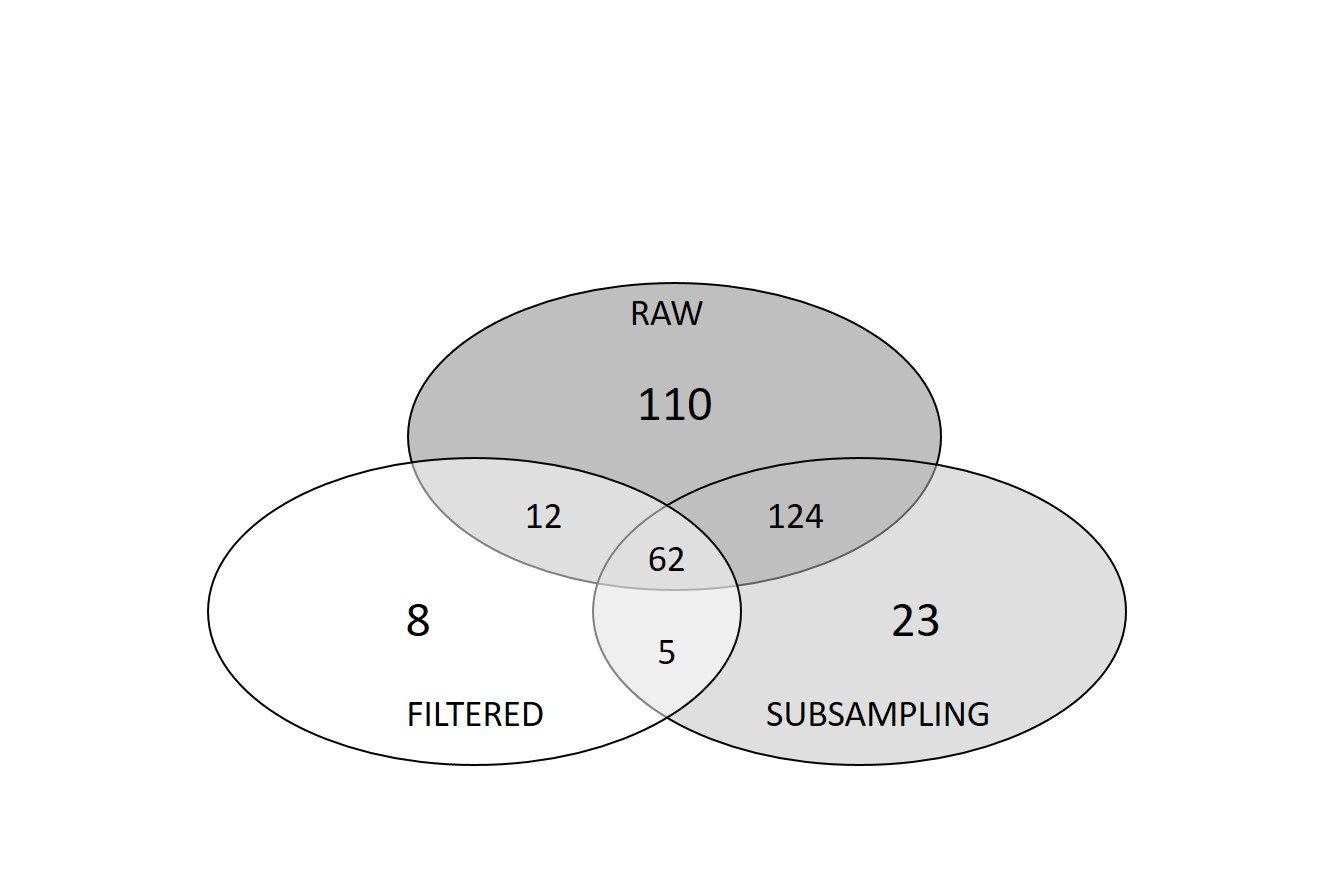

Supplement: S7 Fig — (TIF) [file pone.0193527.s007.tif]

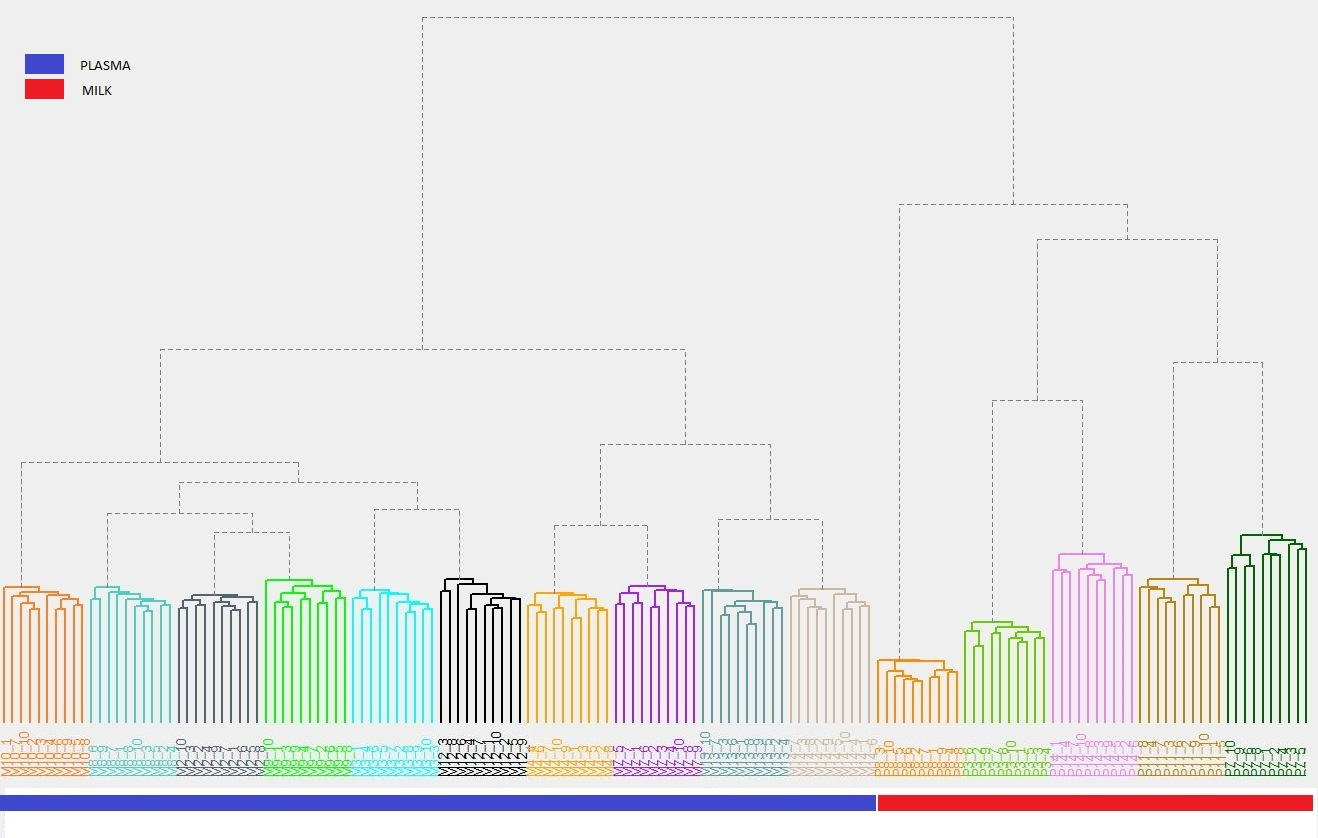

Supplement: S8 Fig — For each sample (10 milk and 5 plasmas) 10 random subsamples of 0.2M reads of miRNA were obtained. Samples classify by biofluid and by sample of origin. Coloured branches represent 10 subsamples of the same sample of origin and bottom boxes the biofluid. (TIF) [file pone.0193527.s008.tif]

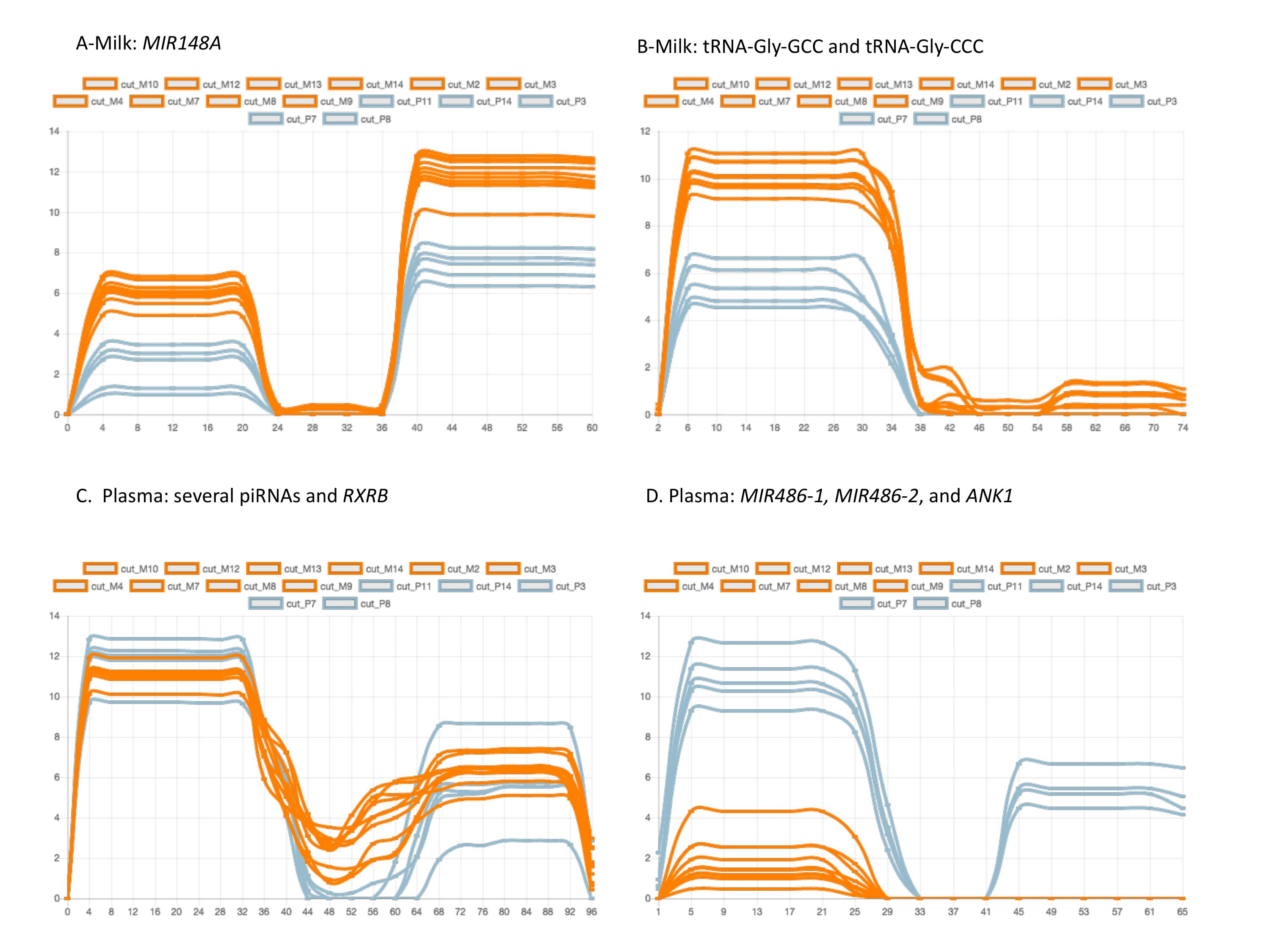

Supplement: S9 Fig — Y-axis represents the abundance profile, and x-axis the positions in the precursor. A) Milk: MIR148A, B) Milk: tRNA-Gly-GCC and tRNA-Gly-CCC; C) Plasma: Several piRNAs and RXRB gene; D) Plasma: MIR486-1, MIR486-2, and ANK1 gene. In orange milk samples, and in blue plasma samples. (TIF) [file pone.0193527.s009.tif]

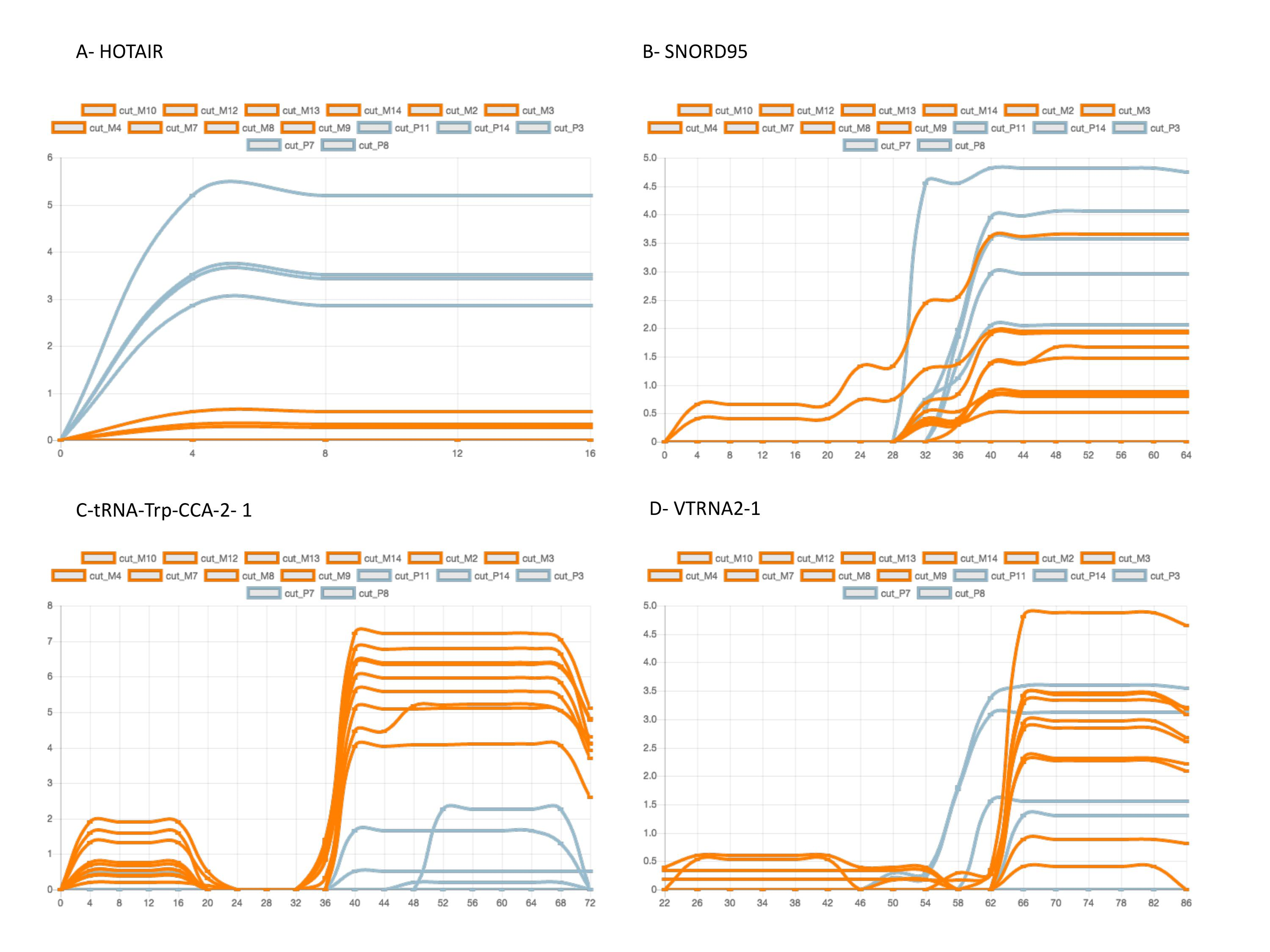

Supplement: S10 Fig — Y-axis represents the abundance profile, and x-axis the positions in the precursor. A) HOTAIR, higher levels in plasma, B) SNORD95, higher levels in plasma; C) tRNA-Trp-CCA-2-1, higher levels in milk; D) VTRNA2-1, higher levels in plasma. In orange milk samples, and in blue plasma samples. (TIF) [file pone.0193527.s010.tif]
